# TRF‐16 Inhibits Lung Cancer Progression by Hindering the N6‐Methyladenosine Modification of CPT1A mRNA

**DOI:** 10.1111/jcmm.70291

**Published:** 2024-12-16

**Authors:** Jiankui Ye, Yu Chen, Zhuowei Shao, Yili Wu, You Li, Shuai Fang, Shibo Wu

**Affiliations:** ^1^ Department of Respiratory Medicine The Affiliated Lihuili Hospital of Ningbo University Zhejiang China; ^2^ Health Science Center Ningbo University Zhejiang China; ^3^ Department of Thoracic Surgery The Affiliated Hospital of Medical School of Ningbo University Zhejiang China

**Keywords:** CPT1A, fatty acid metabolism, IGF2BP1, lung cancer, N6‐methyladenosine, tRFs

## Abstract

Transfer RNA‐derived fragments (tRFs) are a new class of small non‐coding RNAs. Recent studies suggest that tRFs participate in some pathological processes. However, the biological activities and processes of tRFs in lung cancer cells remain mainly unclear. In the present investigation, we employed tRNA‐derived small RNA (tsRNA) sequencing to predict differentially expressed tsRNAs in lung cancer cells, and nine tsRNAs with significant expression alterations were validated using qPCR. Wound healing, colony formation, transwell invasion and CCK‐8 assays were performed to detect the effects of tRF‐16 on cell function. Western blotting evaluated the relationship between tRF‐16 and the IGF2BP1 axis. Our findings demonstrated that tRF‐16 expression was substantially downregulated in lung cancer cells. TRF‐16 could inhibit lung cancer cells' ability to increase, migrate, invade and obtain radiation resistance. Furthermore, tRF‐16 decreases the stability of CPT1A by impairing the binding of IGF2BP1 to CPT1A. As a result, the fatty acid metabolism in lung cancer cells was inhibited. Finally, tRF‐16 also inhibits lung cancer cell proliferation in vivo. This study shows that tRF‐16 plays a crucial regulatory role in the proliferation of lung cancer cells and may represent a novel avenue for their regulation.

## 1 Introduction

Lung cancer is one of the most common cancers in the world and poses a severe threat to human health [1]. About 2.2 million people are diagnosed with lung cancer each year, and 1.75 million die within five years of being diagnosed [1, 2]. When individuals contact their doctor and miss out on surgery, most are diagnosed with advanced‐stage lung cancer [3]. As a result, identifying novel diagnostic biomarkers and treatment targets for lung cancer holds significant clinical relevance [4, 5].

The most recent studies have identified a novel class of non‐coding small RNA known as tRNA‐derived small RNA (tsRNA) [6]. They can be classified into two primary categories by tRNA cleavage: tRNA‐derived fragments (tRFs) and tRNA halves (tRHs or tiRNAs) [7, 8]. It has been observed that tsRNA influences protein translation, RNA transcription or post‐transcriptional regulation to impact the growth, apoptosis and metastasis of tumour cells [9, 10]. For example, tRF‐3017A forms an RNA‐induced silencing complex (RISC) with the protein Argonaute (AGO) to control the tumour suppressor gene NELL2 and enhance the migration and invasion of gastric cancer cells [11]. AS‐tDR007333 enhances malignant tumour proliferation in NSCLC cells by activating MED29 via two mechanisms [12]. tRF‐Glu49 suppresses the proliferation of cervical cancer cells by targeting fibrinogen‐like protein 1 (FGL1) [13]. Moreover, tsRNA possesses potential diagnostic, therapeutic and prognostic significance for different cancers, including colorectal, breast, gastric, nasopharyngeal and ovarian [14, 15, 16].

Fatty acid metabolism provides energy for the growth of cancer cells and is closely connected with cancer signalling pathways [17, 18]. Research indicates that tsRNA indirectly uses multiple signalling pathways to modulate lipid metabolism through interactions with target genes [19, 20, 21]. During fatty acid oxidation (FAO), the carnitine palmitoyltransferase system is responsible for the delivery of long‐chain fatty acids (FA) from the cytoplasm to the mitochondria for oxidation [22]. Carnitine palmitoyltransferase 1A (CPT1A) is a key rate‐limiting enzyme of mitochondrial fatty acid oxidation (FAO) [23, 24]. Inhibition of CPT1A will diminish the transport of fatty acids into mitochondria, compelling the conversion of fatty acids into lipid droplets for storage; thus, the expression of CPT1A is essential for tumorigenesis [25, 26].

N6‐methyladenosine (m6A) is among the most prevalent and abundant modifications of transcripts. The m6A writers, erasers and readers are proteins that add, remove or identify m6A on mRNA or non‐coding RNAs, respectively. These proteins provide crucial biological functions in m6A alterations [27]. Numerous studies have demonstrated that m6A modifications play essential roles in various cancers, often involving the action of m6A modification writers that catalyse the m6A modification of oncogene or tumour suppressor gene mRNAs, followed by the action of readers that recognise these modified mRNAs through molecular biology effects, thereby upregulating or downregulating tumour gene expression [28, 29].

The insulin‐like growth factor‐2 mRNA‐binding protein 1 (IGF2BP1) is an RNA‐binding protein with RNA‐binding domains and m6a‐modified reader [30]. It can regulate the expression of some mRNA targets required for tumour activity and play an essential role in carcinogenesis [31, 32]. Therefore, it is called the ‘carcinomatous’ protein [30, 33]. IGF2BP1 can promote adipocyte differentiation and proliferation [34]. Promoting breast cancer metastases across the USP10/IGF2BP1/CPT1A axis, it may directly identify and bind the m6A site on CPT1A mRNA, hence enhancing its stability [35]. Furthermore, AGR2 and IGF2BP1 accelerate the hypoxic state of kidney cancer cell lines, hence promoting tumorigenesis [36]. As a result, IGF2BP1 has been widely regarded as an oncogene and a cancer therapy target [33, 37].

Our research identified the impact of tRF‐16 on lung carcinoma cells. It can bind to IGF2BP1, decrease the stability of CPT1A and eventually reduce fatty acid metabolism, thus suppressing the proliferation of lung cancer cells.

## 2 Materials and Method

### 2.1 Clinical Specimens and Cell Culture

The research involved the collection of tissue specimens from 105 patients diagnosed with lung adenocarcinoma and 53 healthy persons. All patients had not received chemotherapy or radiotherapy before surgery. Post‐surgery, the excised cancerous and normal tissue specimens were frozen in liquid nitrogen and stored at −80°C until required. The Institutional Review Board (IRB) at Lihuili Hospital, Ningbo, approved this research (Approval No: KY2022SL447–01), and all patients provided signed informed consent. The clinical data and histological information of the tissues were obtained from medical records and pathology reports.

Human normal lung epithelial cells (BASE‐2B) and human lung squamous carcinoma cell lines (PC9, HCC827, A549 and NCI‐H1299) were cultured in DMEM (Gibco, USA). The culture medium for all cells comprised 10% foetal bovine serum (Gibco, USA) and 1% penicillin/streptomycin (PS). The culture was conducted in a 37°C incubator with 5% CO_2_ supplementation.

### 2.2 Cell Transfection

Using genome editing technologies, synthetic analogues and inhibitors of tRF‐16, as well as negative control were developed, and oligonucleotides were then transfected into A549 and H1299 cells to create the overexpression, knockdown and control models for tRF‐16. As directed by the manufacturer, the Lipofectamine 2000 transfection reagent (Invitrogen, America) was used.

### 2.3 TRFs and tiRNAs Sequencing

The A549R and H1299R radiation‐resistant cell lines and their normal controls, A549 and H1299, were used for tRFs and tiRNAs sequencing. RNA sequencing and the construction of the library were performed by KangChen Biotech. The Agilent BioAnalyzer 2100 was used to quantify the sequencing libraries. Standard small RNA sequencing was carried out using the Illumina NextSeq platform with 50 bp single‐end sequencing.

### 2.4 Quantitative Real‐Time Polymerase Chain Reaction (qRT‐PCR)

According to the manufacturer's instructions, the whole RNA from A549/R and H1299/R cells was extracted using a Trizol reagent (Ambion, USA). cDNA was synthesised using a reverse transcription system kit from Genepharma. qRT‐PCR was carried out using SYBR Green (Genepharma, China), and the ACTIN and NAT10 were kept as the endogenous reference on an Agilent Mx3005P fluorescence quantitative PCR instrument to assess the mRNA's relative expression.

### 2.5 Colony Formation Assay

In the log phase, cells (1000/well) were propagated in six‐well plates (NEST, China). Following two weeks, cell colonies were washed three times with 1 × PBS, fixed for 30 min using 4% paraformaldehyde (Solarbio, China), stained for 30 min with 0.5% crystal violet (Solarbio, China) and subsequently counted. Colonies consisting of 50 cells were selected to evaluate the cloning formation rate. Images were obtained with a gel imaging method.

### 2.6 Cell Counting Kit‐8 (CCK‐8) Proliferation Assay

This assay assessed the proliferation capacity of lung cancer cells. Transfected cells (5 × 10^3^/well) were cultured in a 96‐well plate (NEST, China) at various time intervals (0, 24, 48, 72 and 96 h). After adherence, 10 μL of CCK‐8 solution (Dojindo, Japan) was introduced in each well and kept for 2 h at 37°C. The absorbance was acquired via a microplate reader (Potenov, China) at 450 nm.

### 2.7 Wound Healing Assay

The experiment was conducted by culturing cells on a six‐well plate until they reached 90% confluency. Afterwards, uniform scratches were produced using a 200 μL pipette tip, and photos were promptly acquired using an Olympus microscope. The cells were cultured for 24 h in a media without serum before picture acquisition. The scratch width was measured at each time point, and the distance was assessed via Image‐Pro Plus 6.0 software.

### 2.8 Cell Migration Assay

Falcon cell culture inserts were placed in the wells of 24‐well plates to which 500 μL of complete culture medium had been added. 200 μL of DMEM without FBS mixed with 1 × 10^5^ cells were loaded into the upper chamber. Following a 24‐h incubation, the upper chamber was washed with a cotton swab to eliminate cells that had not traversed the membrane. The bottom of the filter was fixed with 4% paraformaldehyde (Solarbio, China) in 0.1 M PBS (Solarbio, China) and stained with a 0.5% crystal violet stain Solution (Solarbio, China). Microscopic photographs were captured in five random fields. To assess the impact on cell migration, we quantified the moving cells after eliminating the proliferating cells. All transwell tests were adjusted for the influence of proliferating cells.

### 2.9 Transwell Invasion Assay

The invasive ability of transfected cells was assessed by the transwell invasion assay. Briefly, cells were collected and resuspended in a serum‐free medium in the matrix gel mixture (BD, USA) coated transwell chambers (Corning, USA) and were inserted into a 24‐well plate containing 20% serum (Gibco, USA). Following 24 h of incubation, the residual cells on the upper surface of the chamber were eliminated using a cotton swab. In contrast, the adherent cells on the lower surface were fixed and stained. The quantity of invading cells was assessed via microscopy and quantified using ImageJ software.

### 2.10 RNA Pulldown Assay

A previous method was utilised to modify an RNA pulldown assay for RNA‐binding proteins. Genepharma synthesised tRF‐16 (sense) and antisense, labelled with biotin. After being reconstituted in 200 μL of binding buffer, the Dynabeads were mixed with either blank control or sense and antisense RNA. The combinations were then allowed to sit at 4°C for 4 h on a shaker. After adding 2 mg of cell‐derived protein lysate (Beyotime, China), the tubes were rotated at 4°C overnight. The RNA–protein complexes were resuspended in PBS and denatured with SDS‐PAGE loading buffer at 99°C for 10 min after being washed three times with PBS on a magnetic separation rack. Proteins were produced using a Fast Silver Stain Kit (Beyotime, China) following electrophoresis on a 7.5% PAGE gel (NCM, China). Significantly, different protein bands were removed and forwarded for investigation by protein mass spectrometry.

### 2.11 RNA Immunoprecipitation (RIP) Assay

A549 and H1299 cells transfected with the pCMV‐FLAG‐IGF2BP1 plasmid were subjected to RIP with EZ‐Magna RIP RNA‐Binding Protein Immunoprecipitation Kit (Millipore, USA). In brief, cells were lysed with RIP lysis buffer containing a protease inhibitor cocktail and RNase inhibitor. 50 μL of protein A/G magnetic beads (Beyotime, China) was washed and mixed with 5 μg of rabbit anti‐DYKDDDDK Tag antibody (Cell signalling technology, USA). Following a 4‐h slow rotation at 4°C, the magnetic beads conjugated to the antibody were isolated and then treated with cell lysates overnight at 4°C. A magnetic stand isolated the antibody–protein–RNA complexes. RNA was extracted and assessed for quality using qRT‐PCR as previously described.

### 2.12 Western Blot Assay

Cellular proteins were acquired with the help of RIPA lysis buffer (Solarbio, China). The device was fixed following the procedure, and the electrophoresis gel was prepared. An equal amount of protein lysate was added to each well in a prearranged order after the buffer, and the proper protein marker was added to the electrophoresis tank. After transfer, the membrane was sealed with skim milk (Solarbio, China) and then kept at 4°C in primary antibodies (anti‐CPT1A, anti‐IGF2BP1 and anti‐ACTIN) (Abcam, England) overnight, followed by secondary antibodies (Proteintech, China) incubation at ambient temperature for 1 h. Finally, the membrane was imaged using the Odyssey system.

### 2.13 Statistical Analysis

tRF and tiRNA sequences were analysed by the R package (version 3.51). Data are presented as the mean ± SD. Using the mean ^Δ^Ct as a reference, triplicate experiment data were normalised before being compared using the two‐tailed *t*‐test. Differences deemed statistically significant were those for which *p* < 0.05. ANOVA was used to compare data when three or more groups were involved.

## 3 Results

### 3.1 TRFs Are Differentially Expressed Between Normal and Lung Cancer Tissue Samples

**FIGURE 1:**
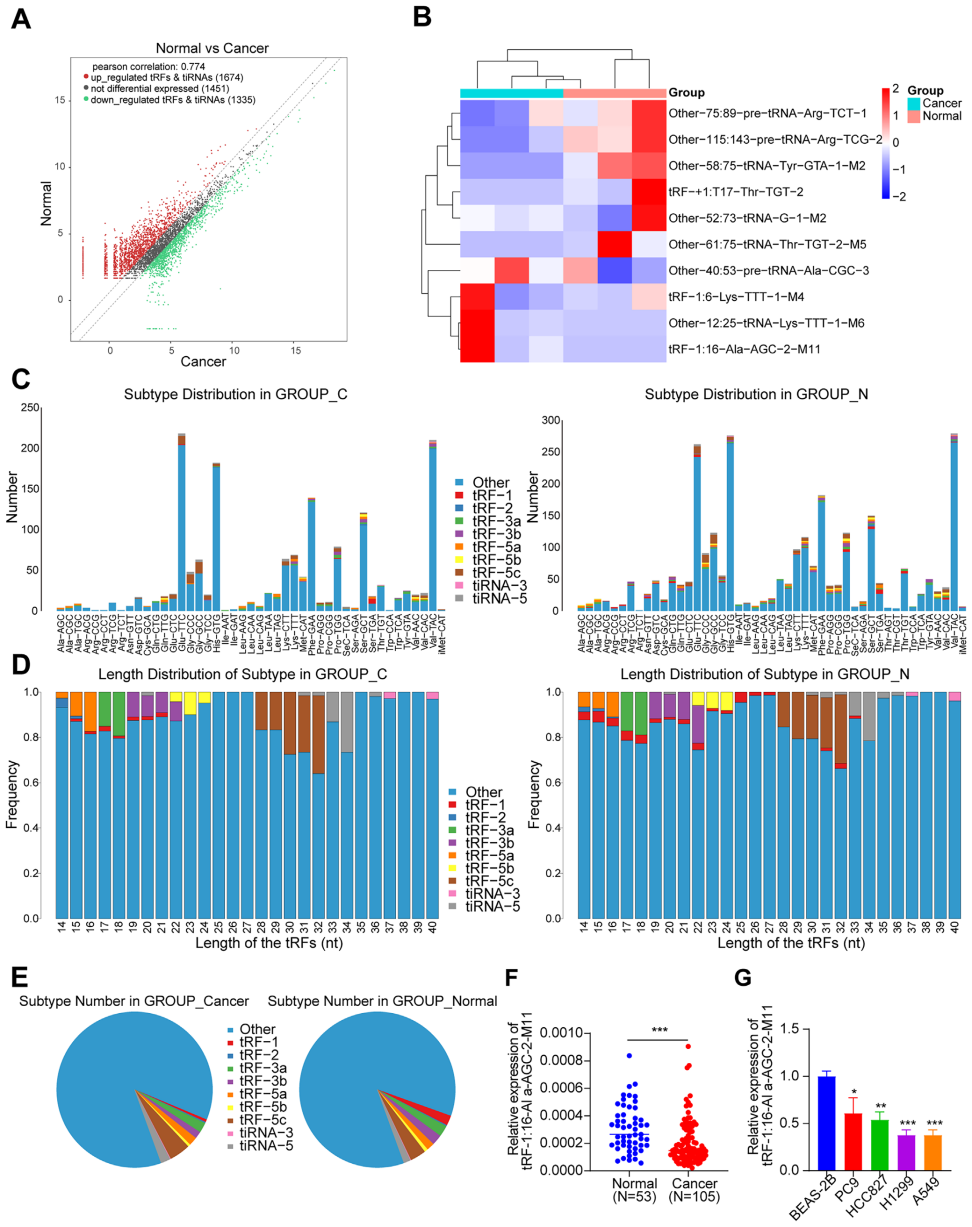
Expression profile of tsRNA in lung cancer. (A) The scatter plot displays differentially expressed tsRNAs in tumour and normal tissues. (B) Hierarchical cluster heatmap of 10 significantly differentially expressed tsRNAs between tumour and normal tissues. (C–D) Subtype distributions and length distributions of different types of tRFs and tiRNAs in tumour tissue and normal tissue. (E) Pie diagram displaying the ratio of each subtype tsRNAs between tumour and normal tissues. (F) Expression of tRF‐1:16‐Ala‐AGC‐2‐M11 in 53 normal tissues and 105 tumour tissues. (G) TRF‐1:16‐Ala‐AGC‐2‐M11 was underexpressed in lung cancer cell lines. *p < 0.05, **p < 0.01, ***p < 0.001.

High‐throughput sequencing analysis of tRFs and tiRNAs in normal and lung cancer tissues revealed significant variations in tRFs and tiRNAs levels between normal and lung cancer tissues, which helped to clarify the differentially expressed tsRNAs in lung cancer tissues. One thousand six hundred and seventy‐four tsRNAs were upregulated, and 1335 were downregulated in the lung cancer tissues than in the normal tissue (Figure 1A). A clustering heatmap of the top 10 most essential differentially expressed tsRNAs is presented in Figure 1B. The distribution of tRFs and tiRNAs in both the tumour group and the surrounding cancer group is comparatively uniform (Figure 1C). Regarding length distribution, several forms of tRFs and tsRNAs exhibit differing lengths, frequently ranging from 14 to 40 nucleotides (Figure 1D). Pie plot analysis revealed that most tRFs were derived from the tRF‐5c; tiRNA series also belonged to tiRNA‐5 (Figure 1E). Furthermore, the sequencing analysis revealed variations in tRFs and tiRNAs between the normal and lung cancer tissue. Afterwards, nine substantially differentially expressed tsRNAs were selected for further experiments (Figure S1A–I). tRF‐+1:T17‐Thr‐TGT‐2 were significantly upregulated in cancer tissues, according to qRT‐PCR validation of the expression of these tsRNAs in cancer tissues and normal tissues. In line with the sequencing findings, tRF‐1:16‐Lys‐TTT‐M4 was also significantly downregulated at the same time. The most crucial differential expression was for tRF‐1:16‐Ala‐AGC‐2‐M11, which was much lower in cancer than in normal tissues (Figure 1F,G). Therefore, tRF‐16 was selected for further investigation.

### 3.2 TRF‐16 Inhibits the Proliferation of Lung Cancer Cells In Vitro

**FIGURE 2:**
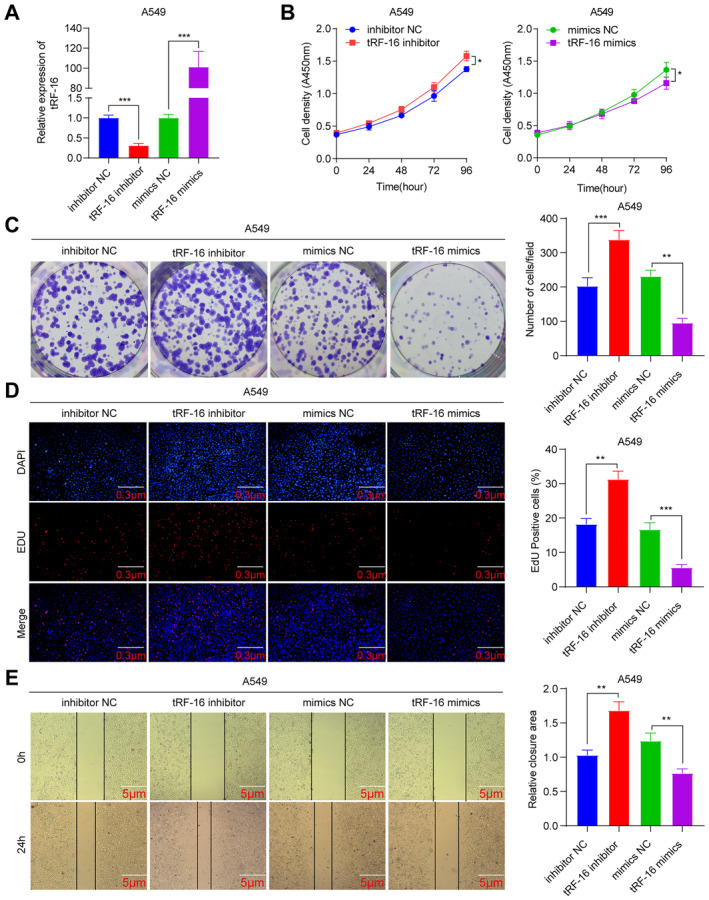
TRF‐16 inhibits the proliferation of lung cancer cells in vitro. (A) qPCR validation of the expression of tRF‐16 in A549 cells after knockdown and overexpression. (B) Cell proliferation was analysed using CCK‐8 assays after knocking down and overexpressing tRF‐16. (C) Colony formation assay indicated cell proliferation after knockdown and overexpression tRF‐16. (D) EDU assay indicated cell proliferation after knockdown and overexpression of tRF‐16. (E) Cell migration was analysed using wound healing assays after knocking down and overexpressing tRF‐16. *p < 0.05, **p < 0.01, ***p < 0.001.

A549 and H1299 cells were transfected individually with tRF‐16 inhibitors and mimics to investigate the relationship between tRF‐16 and lung cancer cells in more detail. These were designated as the inhibition and mimics group, while the wild‐type (A549 and H199) cells without transfection were called the control group. Both groups' tRF‐16 expression was found by qRT‐PCR. It was noted that tRF‐16 expression was much lower in the inhibition group and significantly higher in the mimics group. Furthermore, Figures 2A and Figures S2A show that the difference was statistically significant. The effects of tRF‐16 on lung cancer cell function were determined. The CCK‐8 assay revealed that the cell density in the tRF‐16 inhibition group was significantly greater than in the control group.

In comparison, the cell density in the tRF‐16 mimics group was substantially lower than that of the control group. The results were statistically significant (Figure 2B and Figure S2B). Following that, a colony formation assay was conducted on both the inhibition and mimics groups, revealing that the cell cloning formation capacity of the inhibition group was statistically significantly higher than that of the control group. In contrast, the cell cloning formation ability of the mimics group was statistically significantly lower than that of the control group (Figure 2C and Figure S2C). This suggests that the suppression of tRF‐16 expression can promote the proliferation of lung cancer cells. The EDU results (Figure 2D and Figure S2D) showed that the cell proliferation number of the tRF‐16 inhibition group was substantially increased than the control group, and the mimics group was reduced significantly than the control group, suggesting that tRF‐16 suppresses the proliferation of lung cancer cells. Furthermore, according to the wound healing assay (Figure 2E and Figure S2E), the migration distance of the tRF‐16 inhibition group was substantially elevated than the control group, and the tRF‐16 mimics group was substantially depressed than the control group after 24 h of cultivation as well as the migration rate. This suggests that the suppression of tRF‐16 promotes the migration of lung cancer cells. Inhibition of tRF‐16 expression promotes the proliferation, migration and invasion of lung cancer cells.

### 3.3 TRF‐16 Inhibits Fatty Acid Metabolism in Lung Cancer Cells

**FIGURE 3:**
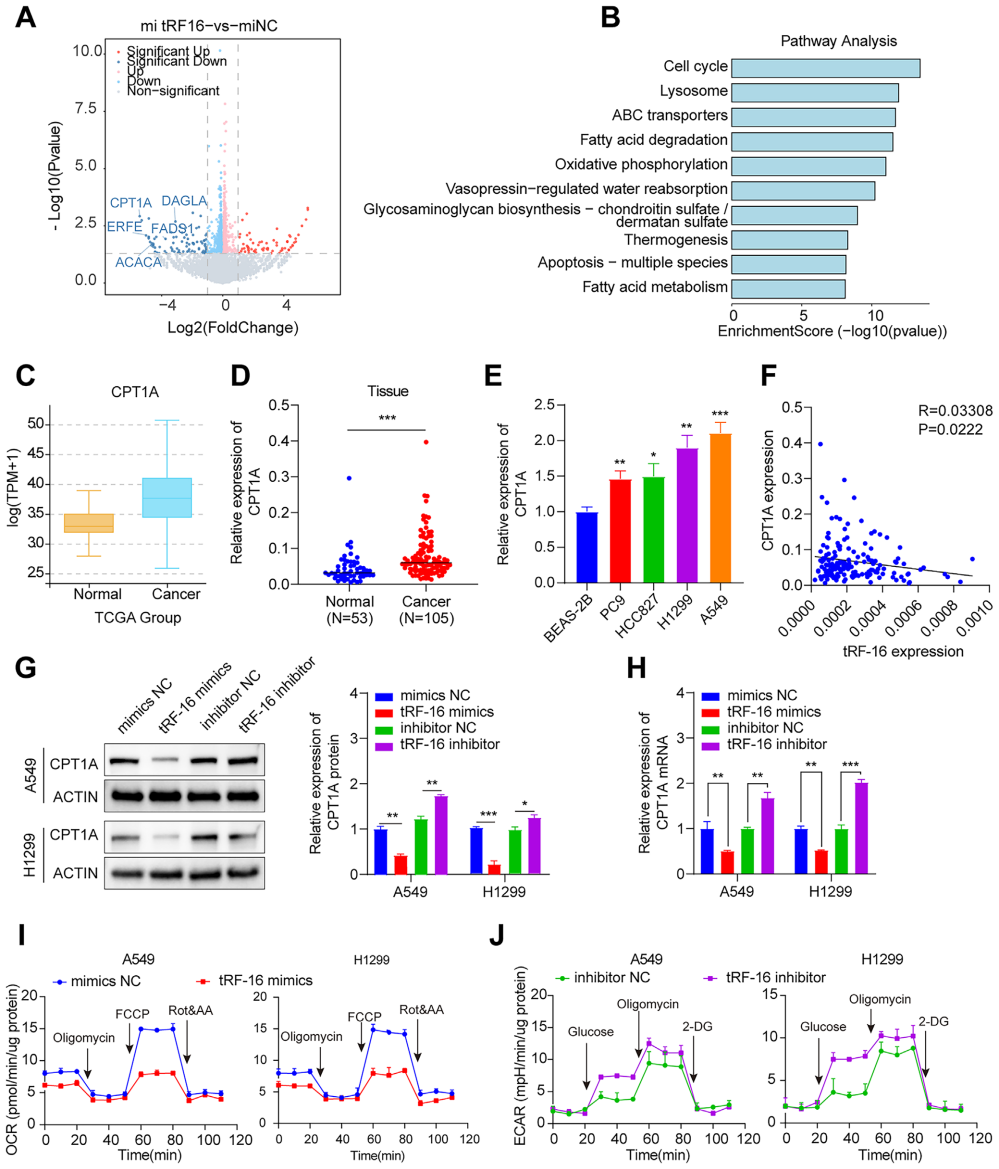
TRF‐16 inhibits fatty acid metabolism in lung cancer cells. (A) RNA‐seq revealed the differentially regulated genes between tRF‐16‐overexpression cells and control cells. (B) KEGG analysis revealed that the target genes of tRF‐16‐overexpression cells were significantly correlated with fatty acid metabolism. (C) CPTIA was significantly upregulated in lung cancer cells compared to normal cells (analysis of TCGA data). (D) CPTIA expression is higher in tumour tissues than in normal tissue by qRT‐PCR. (E) CPTIA expression is higher in tumour cells than in normal tissue by qRT‐PCR. (F) Spearman was used to analyse the relationship between tRF‐16 and CPT1A. (G) Western blot validation of the expression of CPTIA after knockdown and overexpression tRF‐16. (H) qPCR validation of the expression of CPTIA after knockdown and overexpression tRF‐16. (I) Oxygen Consumption Rate (OCR) through overexpression of tRF‐16 in A549 and H1299 cell lines. (J) Extracellular Acidification Rate (ECAR) through knockdown of tRF‐16 in A549 and H1299 cell lines. *p < 0.05, **p < 0.01, ***p < 0.001.

To investigate whether tRF‐16 regulates the downstream genes, RNA sequencing (RNA‐seq) was conducted on lung cancer cells that overexpress tRF‐16 (Figure 3A). KEGG analyses identified the associated signalling pathways of tRF‐16 (Figure 3B). KEGG indicated that the target tRF‐16 genes were markedly enriched in the fatty acid metabolism signalling pathway. Carnitine Palmitoyltransferase type 1 (CPT‐I) is the only enzyme family that catalyses long‐chain fatty acyl‐CoAs to acylcarnitines for translocation across the mitochondrial membrane. The three subtypes of the CPT‐I protein family are CPTIA, CPTIB and CPTIC. CPTIA is the most common member, capable of mitochondrial b‐oxidation, and is linked to developing tumours and drug resistance. Remarkably, we found that in contrast to adjacent tissues and normal lung epithelial cells, lung cancer tissues and cells expressed higher CPT1A mRNA (Figure 3C–E).

Moreover, the analysis showed that tRF‐16 was negatively correlated with CPT1A by Spearman (Figure 3F). To clarify the connection between tRF‐16 and CPTIA, we either overexpressed or knocked down tRF‐16 and evaluated the effect on CPTIA expression. According to our findings, CPTIA expression is promoted when tRF‐16 is absent, while CPTIA expression is reduced when tRF‐16 is overexpressed (Figure 3G,H). To ascertain whether tRF‐16 enhances mitochondrial oxidative metabolism, we analysed the oxygen consumption rate (OCR) in tRF‐16 mimics that were treated. OCR is a biomarker that indicates mitochondrial respiration. Our findings suggested that the treatment with tRF‐16 compounds led to a significantly reduced cellular OCR and a reduction in the basal and maximal respiratory capacity of A549 and H1299 (Figure 3I). We used the extracellular flux method (Seahorse) to measure the extracellular acidification rate (ECAR), a reliable indicator of the rate and capacity of glucose caused by glucose, to examine the effect of tRF‐16 on glycolysis. Surprisingly, the ECAR was significantly increased by the inhibition of tRF‐16 expression (Figure 3J).

### 3.4 TRF‐16 Directly Interacts With IGF2BP1 and Regulates IGF2BP1 Expression

**FIGURE 4:**
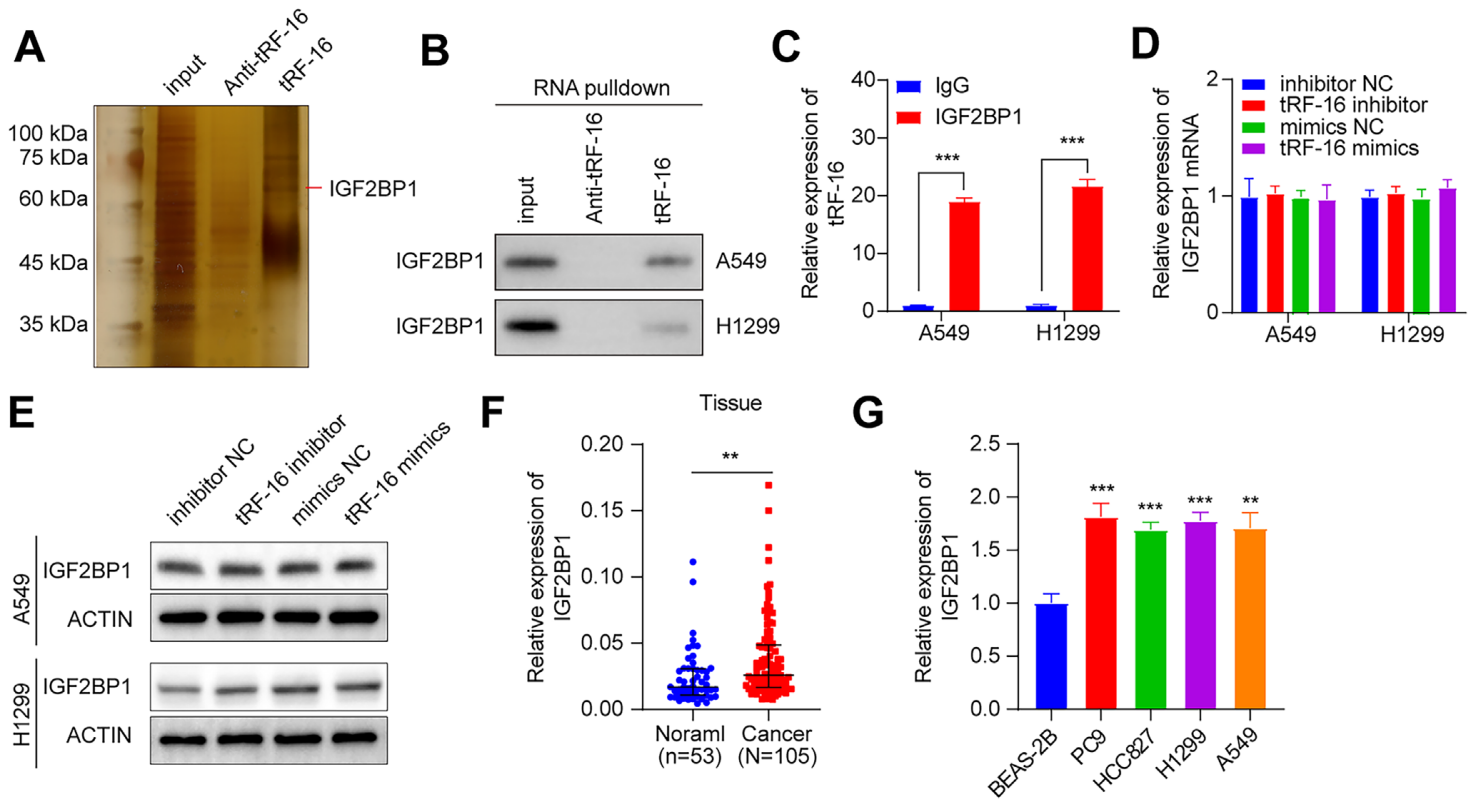
TRF‐16 directly binds to IGF2BP1. (A) RNA pulldown assays and silver SDS‐PAGE gel images are used to select proteins that interact with tRF‐16. (B) The verification of tRF‐16 binds to IGF2BP1 in RNA pulldown assays by western blot. (C) RIP assays confirm the tRF‐16 was detected in the conjugate of IGF2BP1. (D–E) Expression of IGF2BP1 after knockdown and overexpression of tRF‐16 by qRT‐PCR and western blot with its quantification result. (F) IGF2BP1 expression is higher in tumour tissues than in normal tissue by qRT‐PCR. (G) IGF2BP1 expression is higher in tumour cells than in normal tissue by qRT‐PCR. *p < 0.05, **p < 0.01, ***p < 0.001.

To investigate the molecular mechanism behind tRF‐16's function in lung cancer cells, we produced tRF‐16 and its antisense sequence labelled with biotin. We used RNA pulldown experiments in A549 and H1299 cells to identify proteins interacting with tRF‐16. One independent tRF‐16 pulldown assay revealed a ~ 60 kDa band (Figure 4A). IGF2BP1 was verified by pulldown assays and Western blot with the sense sequence but not the antisense sequence (Figure 4B). RNA immunoprecipitation (RIP) assays also proved that tRF‐16 was enriched when an antibody against IGF2BP1 was used for the pulldown assay (Figure 4C). However, tRF‐16 did not influence the IGF2BP1 mRNA level in A549 and H1299 cells by qRT‐PCR and Western blot (Figure 4D,E). In addition, compared with adjacent cancer tissues and normal lung epithelial cells, we observed increased IGF2BP1 mRNA expression in lung cancer tissues and cells (Figure 4F,G).

### 3.5 TRF‐16 Inhibition of CPT1A Stability Receding IGF2BP1 Binding to CPT1A

**FIGURE 5:**
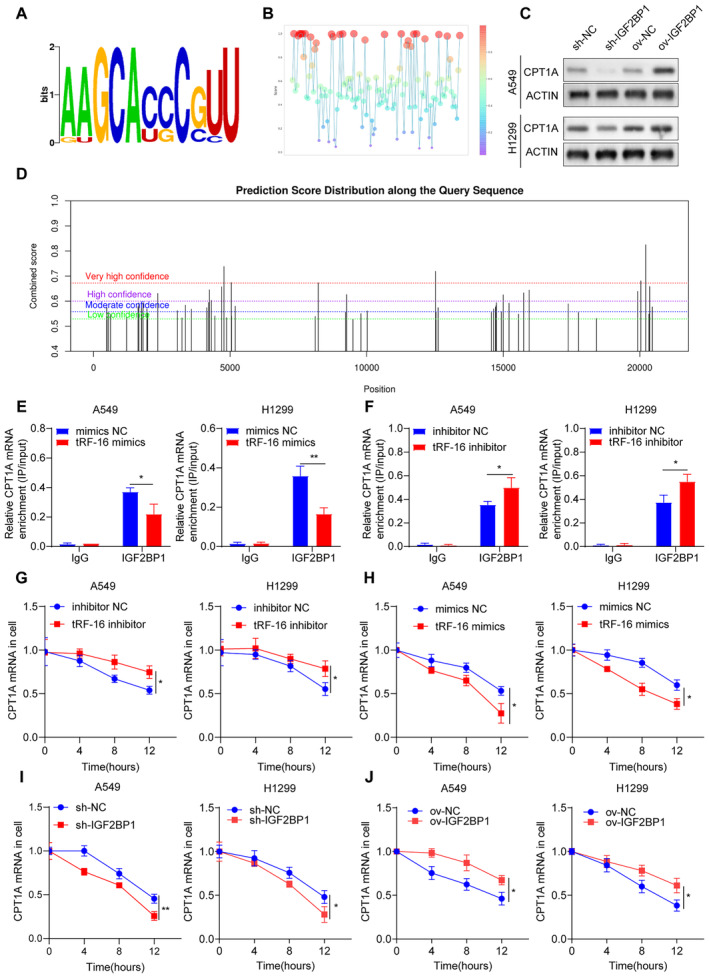
TRF‐16 inhibits the stability of CPT1A by weakening IGF2BP1 binding to CPT1A. (A) Schematic view of the putative IGF2BP1 binding sites. (B) Possibility sites of IGF2BP1 binding on the 3 ‘UTR region of CPT1A, as predicted from RBP suite. (C) Expression of CPT1A after knockdown and overexpression of IGF2BP1 by western blot. (D) Prediction of m6A sites of CPT1A mRNA using the SRAMP database. (E–F) The binding of IGF2BP1 to CPT1A 3 ‘UTR after tRF‐16 overexpression and knockdown by RIP and qRT‐PCR. (G–H) The remaining CPTIA mRNA levels in tRF‐16 knockdown or overexpressed treated with actinomycin D were assessed using qRT‐PCR. (I–J) The remaining CPTIA mRNA levels in IGF2BP1 knockdown or overexpressed treated with actinomycin D were assessed using qRT‐PCR. *p < 0.05.

We hypothesized that additional processes may be involved in the tRF‐16‐associated regulation of IGF2BP1 expression since the tRF‐16‐IGF2BP1 relationship could only partially explain tRF‐16 expression. We discovered potential binding sites for CPT1A were present in the IGF2BP1 promoter using JASPAR and UCSC database studies (Figure 5A). The association between IGF2BP1 and CPT1A mRNA was then predicted using RBPsuite (Figure 5B). Moreover, following IGF2BP1 overexpression and knockdown, CPT1A protein expression dropped (Figure 5C). Several m6A alteration sites in CPT1A mRNA were predicted using the SARMP database (Figure 5D). Moreover, the RIP test was used to confirm the combination of CPT1A mRNA with IGF2BP1 in tRF‐16 inhibition and tRF‐16 mimics (Figure 5E,F). Furthermore, in actinomycin D‐treated cells, the residual CPT1A mRNA level increased after tRF‐16 knockdown, but it dropped following tRF‐16 overexpression, suggesting that tRF‐16 silencing enhanced CPT1A mRNA stability (Figure 5G,H). In actinomycin D‐treated cells, the residual CPT1A mRNA level diminished after IGF2BP1 knockdown and increased following IGF2BP1 overexpression, suggesting that tRF‐16 silencing compromised CPTIA mRNA stability (Figure 5I,J).

### 3.6 TRF‐16 Inhibits Lung Cancer Cell Proliferation In Vivo

**FIGURE 6:**
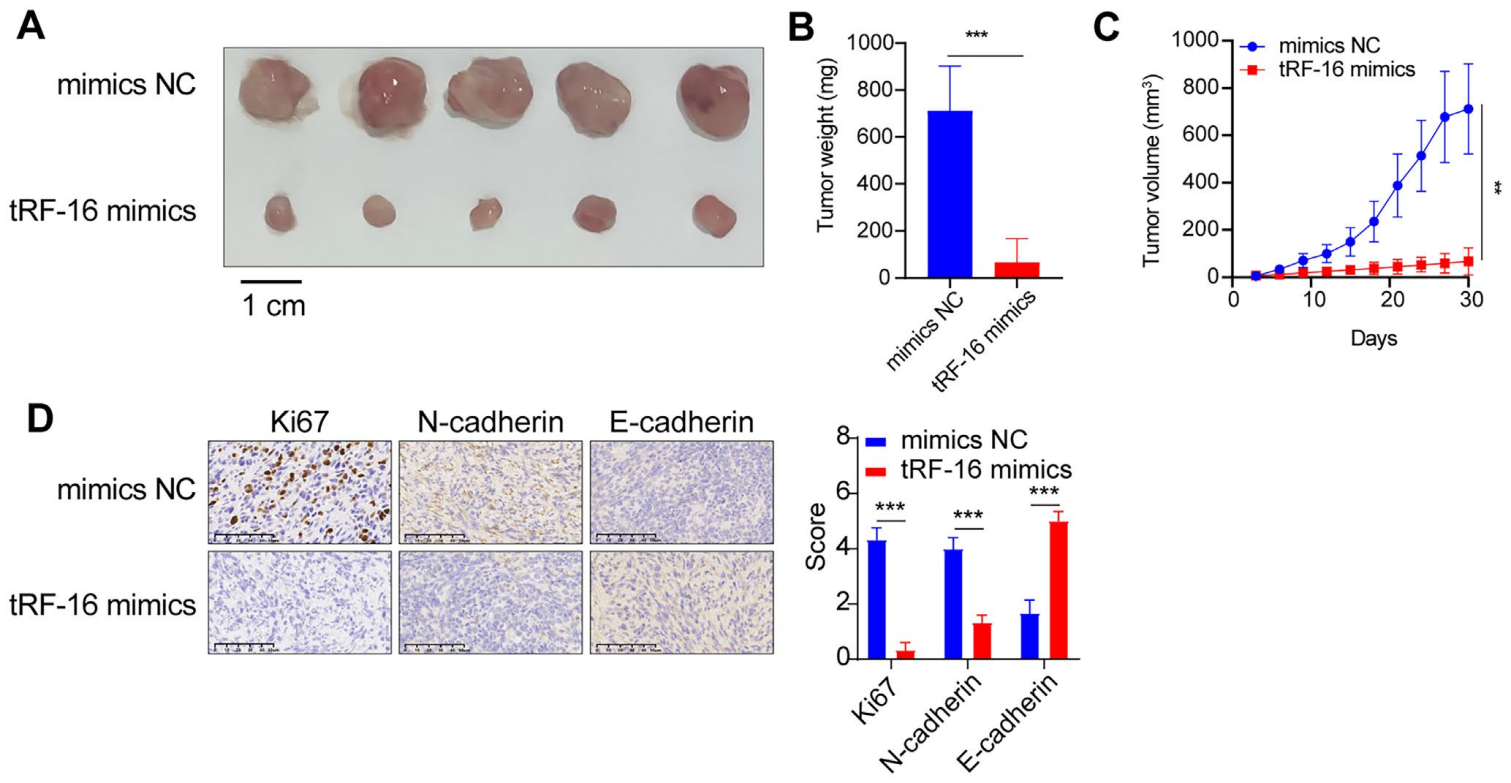
TRF‐16 inhibits lung cancer cell proliferation in vivo. (A) Representative images of the mouse tumours. (B) The mean tumour weight of the mimics group was lower than that of the NC group. (C) The mean tumour volumes of the tRF‐16‐mimics group were significantly smaller than those of the NC group. (D) Representative images of IHC staining. **p < 0.01, ***p < 0.001.

We hypothesized that inhibiting tRF‐16 would have a therapeutic impact on lung cancer cells since tRF‐16 acts as an oncogenic tRF in these cells. For in vivo research, we developed modified tRF‐16 targeted mimics. Figure 6C demonstrates that the tumour volume in the tRF‐16‐mimics group decreased significantly compared to the NC group during the entire study duration. Furthermore, the mean tumour weight in the tRF‐16‐mimics group was considerably (*p* < 0.01) reduced compared to the control groups (Figure 6A,B). Furthermore, the introduction of tRF‐16‐mimics inhibited the expression levels of N‐cadherin, E‐cadherin and Ki‐67 proteins in the xenograft tumour tissues (Figure 6D). As a result, these data indicate that tRF‐16‐mimics may inhibit lung cancer tumour proliferation in vivo.

## 4 Discussion

The present study employed RNA sequencing technologies to clarify the association between tsRNAs and lung cancer cells. The expression of tsRNAs in normal and lung cancer cells exhibited significant variations. In lung cancer cells, 1647 tsRNAs were upregulated, while 1335 were downregulated compared to wild‐type cells. Ten tsRNAs with marked differences were selected for qPCR validation and indicated that tRF‐1:16‐Ala‐AGC‐2‐M11 was substantially downregulated in lung cancer cells, consistent with the sequencing results. Transwell invasion, colony formation, wound healing and CCK‐8 assays demonstrated that suppressing tRF‐16 expression significantly enhanced lung cancer cell proliferation, migration and invasion capabilities. Furthermore, IGF2BP1 directly interacted with tRF‐16, and later cellular tests indicated that tRF‐16 may impede lung cancer cell growth via modulating IGF2BP1 to suppress fatty acid metabolism in lung cancer cells. Currently, little data are available regarding the interaction between RNA and protein. The findings indicate that tRF‐16 may serve as a novel mechanism for regulating lung cancer cell growth and offer innovative strategies for enhancing treatment resistance in lung cancer cells.

In recent years, the continuous development and invention of pertinent research methodologies and technology have led to the emergence of tsRNA in tumour diagnostics. Multiple studies have demonstrated that tsRNA is crucial in tumorigenesis by affecting biological processes like malignant proliferation, invasion and metastasis, angiogenesis, immunological response, resistance to treatment and metabolic reprogramming of tumours [16]. Huang et al. demonstrated that the tRF‐5a fragment modulates the radiation resistance of colorectal cancer cells by targeting MKNK1 [8], whereas tiRNA‐Gly enhances the proliferation and migration of thyroid papillary carcinoma by interacting with RBM17 and causing alternative splicing [38]. tsRNA has a critical role in neurological conditions, metabolic problems and cancer. According to a recent study, tRFGluTTC prevents the development of three T3‐L1 precursor adipocytes into adipocytes [39]. The current investigation identified a distinct tsRNA that suppresses fatty acid metabolism in cancer cells, potentially operating via a novel mechanism.

In both embryonic and tumourous tissues, IGF2BP1 is widely and effectively expressed. Numerous associated studies have reported the augmentation or resynthesis of IGF2BP1 expression in human tumours and animal models, indicating that IGF2BP1 is crucial for the growth of tumour tissue [40]. IGF2BP1 increases the growth, survival, invasion and non‐adhesion of tumour cells in the majority of cancers, as well as their resistance to treatment [38]. However, IGF2BP1 exhibits tumour‐suppressive characteristics in colon stromal cells and breast cancer. It has been discovered that IGF2BP1 regulates genes linked to the metabolism of fatty acids [34, 41]. The current investigation revealed that tRF‐16 promotes the proliferation of lung cancer cells through its interaction with IGF2BP1, indicating that IGF2BP1 could serve as a significant target for cancer treatment.

CPT1A serves as a rate‐limiting enzyme in mitochondrial fatty acid oxidation (FAO) and is a vital component in numerous cancers. CPT1A enhances the translocation of long‐chain fatty acids from the cytoplasm to the mitochondria for oxidation, initiating cyclical processes that result in fatty acid shortening [42]. These processes produce NADH and FADH2 in each cycle, which subsequently yields ATP upon entering the electron transport chain and are directly associated with the metabolism of cancer cells [22]. CPT1A has been documented to be upregulated in various cancers, including breast cancer [43]. In addition, IGF2BP1 can directly identify, bind and stabilise the m6A region on CPT1A mRNA, which in turn mediates IGF2BP1‐induced breast cancer metastasis [35]. This study showed that tRF‐16 can decrease IGF2BP1's ability to bind to CPT1A and decrease CPT1A's stability, which can impact lung cancer cells' fatty acid metabolism and promote both in vivo and in vitro growth.

In conclusion, the current investigation discovered how tRF‐16 affects lung cancer cells. TRF‐16 can bind to IGF2BP1, which weakens CPT1A's stability and has an effect. These tRF‐16‐related discoveries offer novel insights into the molecular relationships between tRNA fragments and RNA‐binding proteins, which could aid in the development of accurate methods for tumour detection and therapy.

## Author Contributions

Conceptualization: Jiankui Ye and Shibo Wu. Methodology: Shuai Fang and Yu Chen. Validation: Zhuowei Shao and Yili Wu. Formal analysis: Jiankui Ye. Investigation: Zhuowei Shao, Yili Wu and You Li. Resources: Shibo Wu. Data curation: Yu Chen. Writing – original draft preparation: Jiankui Ye, Yu Chen and Shuai Fang. Writing – review and editing: Jiankui Ye and Shibo Wu. Visualisation: Yu Chen. Supervision: Shibo Wu. Project administration: Jiankui Ye. Funding acquisition: Shibo Wu. All authors have read and agreed to the published version of the manuscript.

## Ethics Statement

This study was approved by the Medical Ethics Committee of the Affiliated Hospital of Medical School of Ningbo University (No. KY2022SL447–01).

## Conflicts of Interest

The authors declare no conflicts of interest.

## Supporting information


**FIGURE S1.** Expression of tsRNA in lung cancer.

**FIGURE S2.** TRF‐16 inhibits lung cancer cell’s ability to proliferate in vivo.

Data S1.

## Data Availability

Data supporting the reported results are available on request from the authors.
