# Supplementary figures and images for: TRF‐16 Inhibits Lung Cancer Progression by Hindering the N6‐Methyladenosine Modification of CPT1A mRNA
Source: J Cell Mol Med. 2024 Dec 16;28(24):e70291. doi: 10.1111/jcmm.70291 (PMC11647991; doi:10.1111/jcmm.70291)

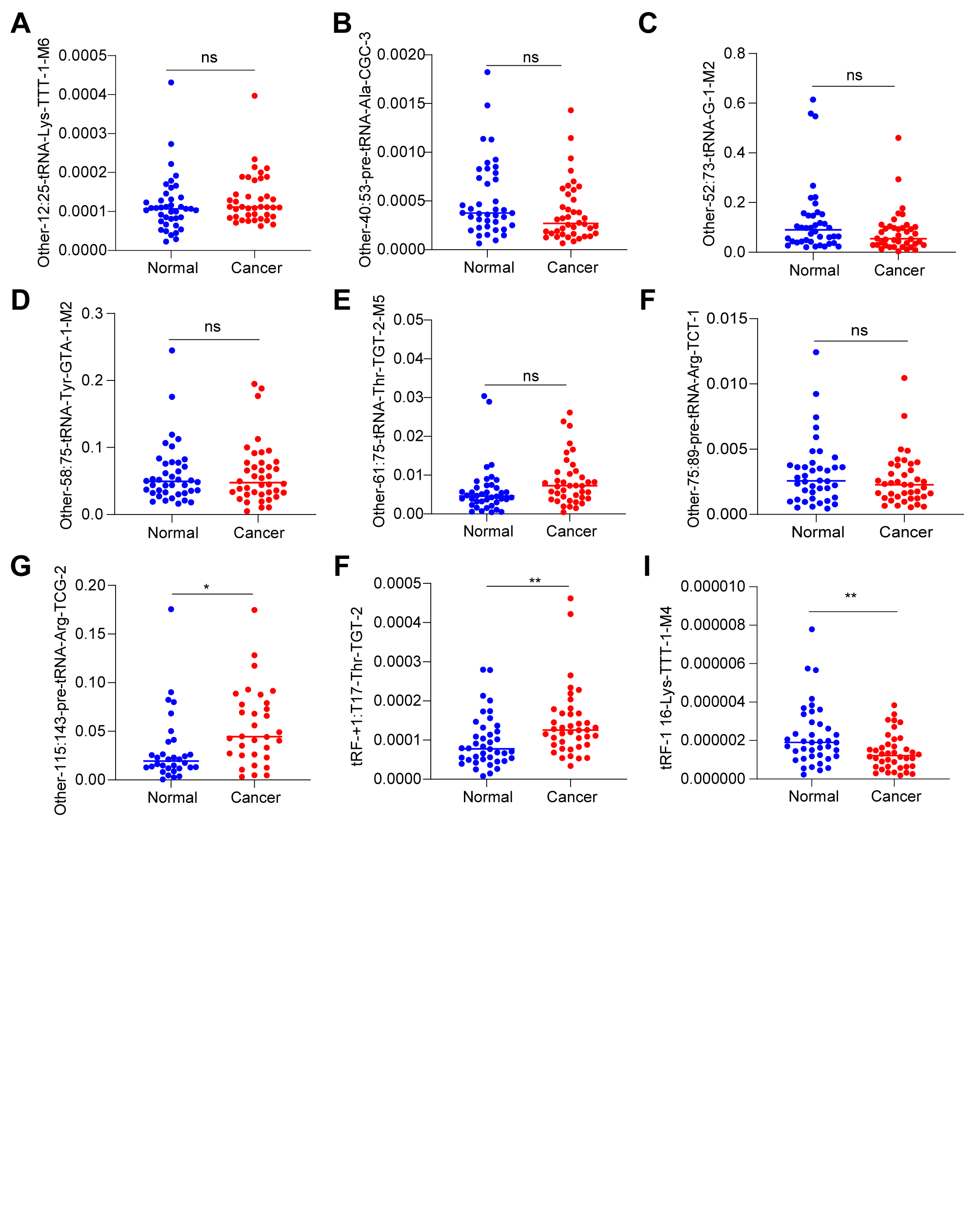

Supplement: Supplementary file 1 — FIGURE S1. Expression of tsRNA in lung cancer. [file JCMM-28-e70291-s003.tif]

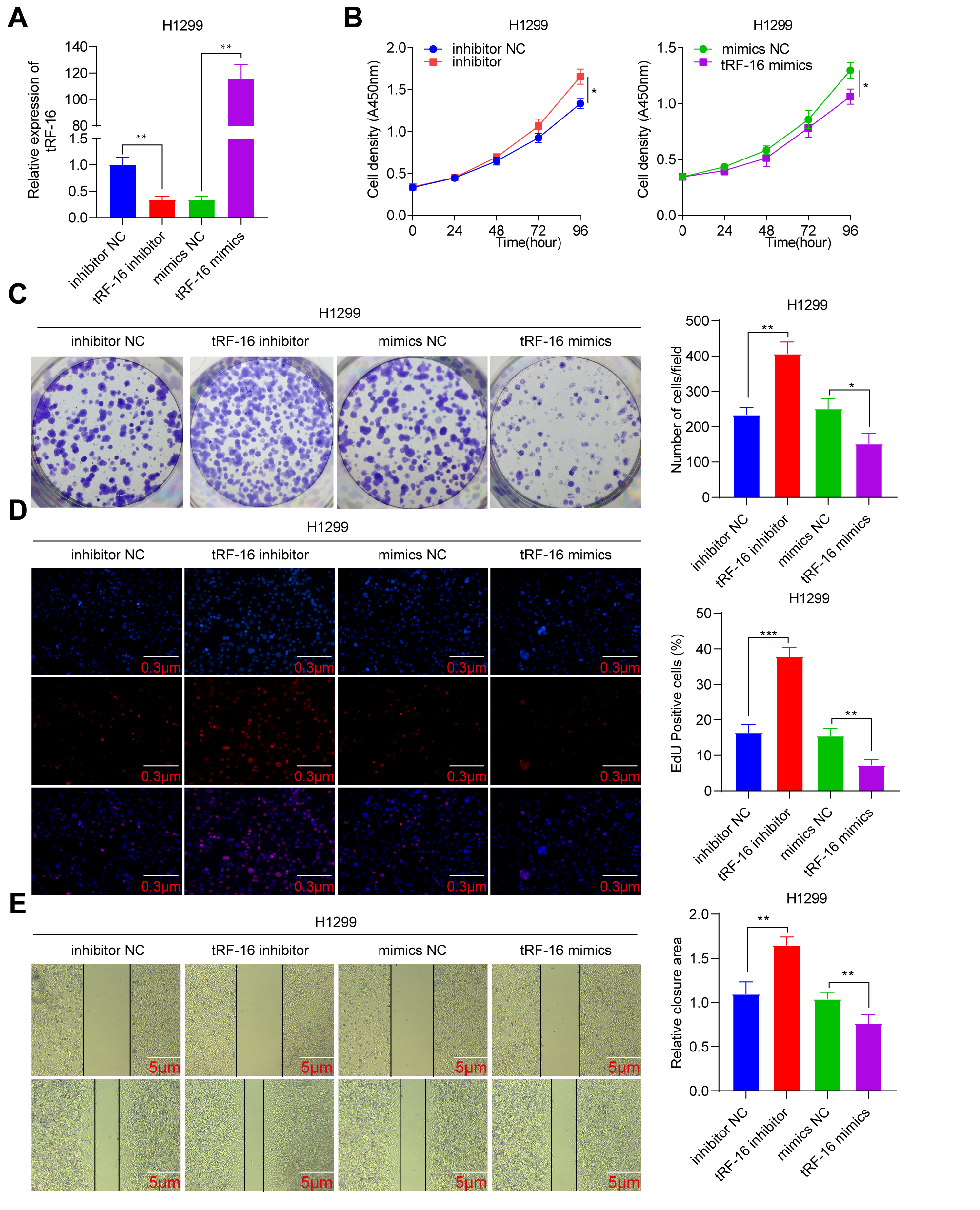

Supplement: Supplementary file 2 — FIGURE S2. TRF‐16 inhibits lung cancer cell’s ability to proliferate in vivo. [file JCMM-28-e70291-s001.tif]

Fig 3G

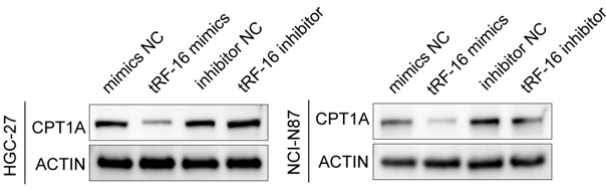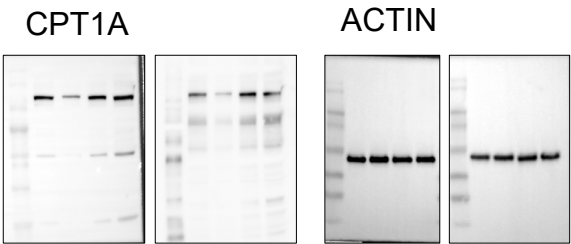

Fig 4B

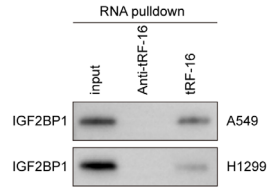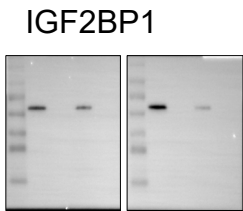

Fig 4B

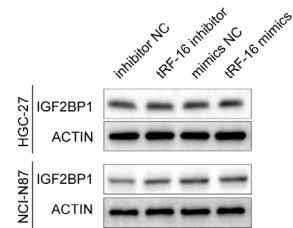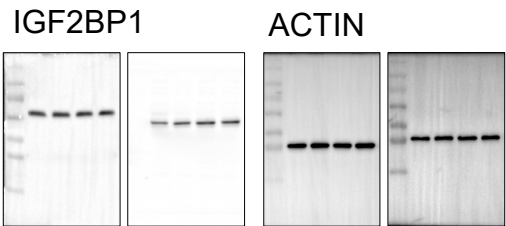

Fig 5C

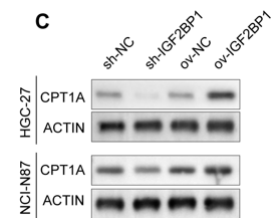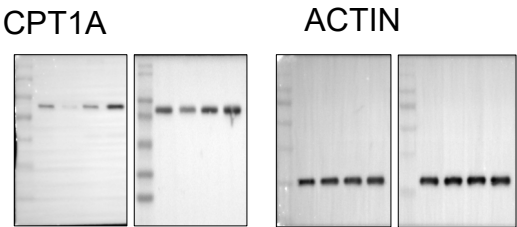

Supplement: Supplementary file 3 — Data S1. [file JCMM-28-e70291-s002.pdf]
